# Molecular Characterization and Phylogenetic Analysis of Honeybee (*Apis mellifera*) Mite-Borne Pathogen DWV-A and DWV-B Isolated from Lithuania

**DOI:** 10.3390/microorganisms12091884

**Published:** 2024-09-13

**Authors:** Paulina Amšiejūtė-Graziani, Vaclovas Jurgelevičius, Simona Pilevičienė, Žygimantas Janeliūnas, Jana Radzijevskaja, Algimantas Paulauskas, Česlova Butrimaitė-Ambrozevičienė, Ingrida Jacevičienė

**Affiliations:** 1 Faculty of Natural Sciences, Vytautas Magnus University, Universiteto str. 10, Akademija, Kaunas District, LT-44248 Kaunas, Lithuania; vaclovas.jurgelevicius@vdu.lt (V.J.); jana.radzijevskaja@vdu.lt (J.R.); 2 National Food and Veterinary Risk Assessment Institute, J. Kairiukscio str. 10, LT-08409 Vilnius, Lithuania; simona.pileviciene@nmvrvi.lt (S.P.); zygimantas.janeliunas@nmvrvi.lt (Ž.J.); ceslova.ambrozeviciene@nmvrvi.lt (Č.B.-A.); ingrida.jaceviciene@nmvrvi.lt (I.J.)

**Keywords:** honeybees, Lithuania, deformed wing virus, *Varroa destructor* mites, DWV-A, DWV-B

## Abstract

Deformed wing virus (DWV) is known as one of the main viruses that affect honeybees’ health all around the world. The virus has two widespread genotypes, DWV-A and DWV-B (VDV-1), transmitted mainly by *V. destructor* mites. In this study, we collected honeycombs with covered broods from 73 apiaries in eight Lithuanian regions and initially investigated the prevalence of *V. destructor* mites. Mites were collected from May to the end of July in 2021 from 124 hives. The prevalence of *V. destructor* infestations in beehives reached 30% and 63% in investigated apiaries. The presence of DWV-A and DWV-B pathogens in mites and broods was examined by RT-qPCR targeting the *CRPV-capsid* region. The molecular characterization of the virus in mite samples was based on sequence analysis of the RNA-dependent RNA polymerase (*RdRp*) region. In addition, leader polypeptide (*LP)*, structural protein (*Vp3)*, *Helicase*, and *RdRp* genes were used for phylogenetic characterization of dual infection. The prevalences of DWV-B in mites and broods were 56.5% and 31.5%, respectively, while DWV-A was detected in 12.9% of mite samples and 24.7% of brood samples. Some of the examined mite samples harboured dual virus infections. Our findings showed that bee colonies from the same apiary were not always infected by the same viruses. Some bee colonies were virus-free, while others were highly infected. Phylogenetic analysis of 21 sequences demonstrated the presence of highly variable DWV-B and DWV-A genotypes in Lithuania and possible recombinant variants of the virus. This study represents the first molecular characterization of mite-borne pathogens hosted by honeybees (*Apis mellifera*) in Lithuania.

## 1. Introduction

The European honeybee (*Apis mellifera*) is known as an important pollinator with huge ecological and agricultural importance throughout the world [1,2,3]. However, parasites and pathogens are strongly implicated in honeybee colony losses [4]. Losses of honeybee colonies are mostly associated with the mite *Varroa destructor*, which feeds on the internal tissues of larvae, pupae, and adult honeybees [2,5,6,7,8,9,10]. Varroa-infested bees usually lose weight and have a shortened life span, and the colony may perish [2]. The mite as a vector can transmit the Acute Bee Paralysis Virus (ABPV), Israeli Acute Bee Paralysis Virus (IAPV), Kashmir Bee Virus (KBV), Sacbrood Virus (SBV), and Deformed Wing Virus (DWV) [11,12,13]. Viruses are able to replicate their genetic material in the mite as well [11,14].

DWV has been assigned to the genus *Iflavirus* in the picorna-like family *Iflaviridae* [15,16]. The single-stranded positive-sense RNA genome has a single open reading frame (ORF) [17]. The DWV infection can cause symptoms like shrunken, crumpled wings; reduced body size; discoloration in adult bees, and reduction in the life span [6,18]. The mite-mediated DWV transmission allows for at least one of the three DWV genotypes or master variants (types A, B, or C) to be selected in honeybees [19]. A fourth variant, DWV-D, has recently been recovered from exhumed Egyptian honeybees collected in the 1970s. It is assumed that DWV-D has been replaced by DWV-A [20,21]. DWV was initially classified as being composed of two master variants: type A, which consists of DWV and Kakugo virus (KV) [16,22], and type B, which was first isolated from *V. destructor* mites in 2001 [14], and originally was named Varroa Destructor virus-1 (VDV-1) [23,24]. DWV-A and DWV-B have 84% nucleotide identity in their genomes [14,25]. DWV type-C is the last master variant of the DWV species complex and is phylogenetically distinct from both A and B types [26]. DWV-B infections associated with parasitic mites can cause the most severe symptoms [27,28]. However, both viruses, DWV-A and DWV-B, have shown the ability to infect both *Varroa* mites and bees on several continents [29].

DWV-A and DWV-B are widespread viruses and are likely to significantly impact honeybee colony health [21]. However, DWV-B has spread around the world more recently than DWV-A [14,30] and is replacing the DWV-A strain in several countries. *V. destructor*-mediated transmission of DWV selects highly virulent strains and decreases overall virus population diversity [31,32]. DWV-B and its recombinants are more virulent than DWV-A strains [33]. *V. destructor* mite infestation, virus transmission, nucleotide, or strain variation can influence the severity of DWV [34]. 

DWV infection rates are reduced by controlling mite infestations using chemical agents or naturally removing infected colonies. Both methods can protect the colony from winter mortality or at least reduce mortality risks [35,36]. Bees’ immune systems also play a vital role in their health and resistance to other bee diseases in the absence of *V. destructor* [10,37].

Despite the worldwide distribution of DWV and the frequency of virus strains, only a few studies have focused on the genetic diversity of these viruses. Especially little is known about the prevalence and genetic diversity of DWV-B (VDV-1) in Lithuania. Direct sequencing of the amplicons and phylogenetic analyses of the sequences provide insights into the genetic relationships between different virus strains. In this study, we report that DWV-B is currently widespread in Lithuania alongside DWV-A. The nucleotide sequences of DWV-B from Lithuania were analyzed and compared with those reported previously from other countries. We also analyzed the phylogenetic relatedness of the partial nucleotide sequences of structural RNA-dependent RNA polymerase region (*RdRp*) of the DWV-A and DWV-B isolates from the different regions of Lithuania to assess the genetic relationship between DWV-A and DWV-B strains of various geographic origins. Several samples with dual viral infection were also molecularly characterized by using fragments of the *LP*, *VP3*, *Helicase*, and *RdRp* genes.

## 2. Materials and Methods

### 2.1. Sample Collection

Honeycombs with covered broods were collected from eight regions of Lithuania in 2021 from May to the end of July. A total of 73 apiaries and 413 honeybee colonies from 37 territories of Lithuania were investigated to detect the presence of *V. destructor mites* and to determine the prevalence of mite infestation. Samples from the state health-monitoring program of bee colonies were provided by the National Food and Veterinary Risk Assessment Institute (NFVRAI) of Lithuania and used for the research. The examination was carried out by the wet method, standardized in the bacteriology unit of NFVRAI: the brood was removed from the comb by using a honeycomb uncapping fork, then broods and honeycomb pieces were placed in a container filled with warm (50–60 °C) water. The container was tightly covered, placed in a shaker, and shaken for 10–15 min. A double honey strainer hung above the sink, and a filter paper or disposable towel was placed on the bottom strainer. The shaken contents of the container were sieved through a double sieve, the upper part of which collected brood and pieces of honeycomb, and the lower part trapped mites. Then, the numbers of mature and immature (larvae and nymphs) *V. destructor* mites were counted.

### 2.2. Extraction of Viral RNA

Broods collected from the same apiary were pooled in one 10 mL tube. Later, the abdominal parts from 5 to 10 broods were transferred into a 2 mL tube with beads. Mites collected from the same bee colonies were also pooled into one tube with beads. Pooled samples of broods and mites were homogenized in 1 mL of sterile PBS buffer with a TissueLyser II device (Retsch, Haan, Germany, 2008). The homogenates were centrifuged at 10,000 rpm, and the supernatant was used for RNA extraction with the RNeasy Mini Kit (Qiagen, Hilden, Germany) according to the manufacturer’s instructions, with minor adjustments. Total RNA was eluted in 60 µL of elution buffer and stored at −20 and −70 °C until use.

### 2.3. RT-qPCR Amplification of Viral RNA

Reverse transcription–quantitative polymerase chain reaction (RT-qPCR) was used to detect the prevalence of DWV-A and DWV-B infections. The reaction was carried out in a volume of 25 µL comprising 20 µL of the reaction mixture and 5 µL of the sample. Negative (DNase/RNase-free water as template) and positive controls were used in each PCR run. As positive controls for DWV-A and DWV-B detection and confirmation, the pC1 clone (GenBank accession: AY292384, position 4240–4659) and pFab1 clone (GenBank accession: AY254569.2, position 2551–4573) were used, respectively. Amplification was performed using the following thermal profile: 10 min at 45 °C and 10 min at 95 °C, followed by 40 cycles of 30 s at 95 °C, 45 s at 55 °C, 45 s at 72 °C, and a final extension step for 5 min at 72 °C. To minimize the risk of false positives in mite samples, an upper cycle threshold (Ct) of 30 was applied for the detection of DWV-A and 35 Ct for the detection of DWV-B, while an upper Ct of 38 was used for DWV-A and DWV-B detection in brood samples. The 7500 ABI real-time PCR system was used to perform the reaction. The gene-specific primer sets, probes, locations of the primers, and the expected product sizes are shown in Supplementary Table S2. The following research was performed only with mite samples.

### 2.4. Preparation for Nucleotide Sequencing 

For further analysis, 22 samples, which were collected in various parts of Lithuania and showed a large amount of virus particles in the samples (Ct < 30), were selected. For sequencing, the RNA-dependent RNA polymerase (*RdRp*) region was used. The amplification of *RdRp* fragment of the virus was performed using SYBR Green mix (QIAGEN, Germany) and a Rotor-Gene Q real-time PCR system (QIAGEN, Germany) with the following thermal profile: 30 min at 50 °C and 15 min at 95 °C, followed by 40 cycles of 30 s at 94 °C, 30 s at 55 °C, 30 s at 72 °C, and a final extension step for 10 min at 72 °C. The gene-specific primer sets, locations of the primers, and the expected product sizes are shown in Supplementary Table S3. Post-amplification melting curve analysis was used to check for non-specific amplification (50–90 °C with increment of 0.5 °C s^−1^). For confirmation, amplification products were analyzed by the capillary electrophoresis QIAxcel Advanced System (Qiagen, Hilden, Germany) using the QIAxcel DNA High-Resolution Kit, QX Alignment Marker 15–1000 bp, QX Size Marker 25–500 bp, OM500 separation method, and a sample injection time of 10 s. The Biocalculator QIAxcel ScreenGel Software 1.5.0 also measured the fragment length and produced a virtual gel image for each run (Supplementary Figure S1).

Dual infections were observed by using the *RdRp* gene. Nine mite samples were selected and analysed using leader polypeptide (*LP*), structural protein (*Vp3*), *Helicase*, and RNA-dependent RNA polymerase (*RdRp*) genes. The gene-specific primer sets, thermal profiles, and expected product sizes are shown in Supplementary Table S4. The target virus gene amplification was performed using One-Step RT-qPCR Master Mix (Oasig, Middlesex, UK) and the Rotor-Gene Q real-time PCR system (QIAGEN, Germany). Thermal profiles were selected during experimental studies. For confirmation and fragment quality determination, amplification products were analysed using gel electrophoresis, and the five highest-quality samples were selected for sequencing.

PCR products were purified and prepared for further analysis using a BigDye XTerminator^TM^ Purification Kit (applied biosystems, Waltham, MA, USA) according to the manufacturer’s instructions. The products were sequenced using a SeqStudio™ Genetic Analyzer (applied biosystems).

### 2.5. Phylogenetic Tree Construction and Analysis of DWV-A and DWV-B Sequences

The generated sequences were compared with sequences in GenBank using BLAST (Basic Local Alignment Search Tool) on the NCBI (National Centre for Biotechnology Information) [38]. A phylogenetic tree for the *RdRp* region (296 bp) was constructed using twelve DWV-B and nine DWV-A genotypes isolated from *V. destructor* mites in the present study and those previously reported in other countries: DWV-B genotypes from the Netherlands (MN538210.1, AY251269.2, MT415952.1), Czech Republic (OL803827.1), Italy (MT747987.1), Israel (JF440525.1), Austria (OL803829.1, OL803828.1), and Belgium (KX783225.1) and DWV-A genotypes from Sweden (MH267696.1, MN746311.1, MT636326.1, MT636324.1, MZ867710.1), Czech Republic (OL803824.1), Italy (KF311109.1, MH223316.1), China (MZ821836.1), Spain (MT096529.1), UK (GU109335.1), Syria (MW265929.1), USA (MG831201.1), and Ireland (MZ867714.1).

Five out of nine samples with dual viral infection were successfully amplified using different primers for four genes of DWV-A and DWV-B. A total of 34 good-quality sequences of *LP*, *Vp3*, *Helicase*, and *RdRp* genes whose lengths varied from 208 to 533 bp were generated (Table 1), and, together with the corresponding sequences from GenBank, were included in the phylogenetic analysis.

Multiple sequence alignment was performed using the ClustalW algorithm, phylogenetic trees were constructed with the MEGAX package [39] using the maximum likelihood method with the Tamura–Nei model [40], and bootstrap values were based on 1000 replicates.

## 3. Results

### 3.1. Prevalence of Mites and Viruses

In total, 1250 *V. destructor* mites from 124 hives were collected. The collected number of mites varied from 1 to 322 in a hive. *V. destructor* mites were found in 63% (46/73) of investigated apiaries and 30% (124/413) of bee colonies (Supplementary Table S1).

Initial screening of viruses by RT-qPCR targeting the *CRPV* capsid region (Supplementary Table S2) showed that the DWV-A virus was detected in 12.9% (16/124) of *V. destructor* mites collected from coated brood samples (Ct < 30), while DWV-B was detected in 56.5% (70/124) of samples (Ct < 35). In five mite samples, dual DWV-A and DWV-B infection was detected.

The prevalence of DWV-B in brood samples was 31.5% (23/73), which was almost half the rate observed in mite samples. Conversely, DWV-A infection in brood samples was 24.7% (18/73), twice as high as that in mite samples. Coinfection with both viruses was detected in six brood samples. Viruses were found in brood samples collected from both mite-infested and Varroa free apiaries. There have also been cases when mites were found to be infected, but no infection was detected in the bee brood.

### 3.2. Phylogenetic Analysis of DWV-A and DWV-B RNA-Dependent RNA Polymerase Region Genotypes

For further analysis, we selected 22 samples of *V. destructor* (Supplementary Figure S1) collected in regions with the highest mite infestation rates. Samples were strongly (<25 Ct) and weakly (25 < Ct < 35) positive when using RT-qPCR with both sets of primers mentioned above (Supplementary Tables S2 and S3). After applying capillary electrophoresis, we selected the samples that showed the highest quality of the target virus and were true-positive for DWV-B and DWV-A infection (Supplementary Figures S1 and S2).

**Figure 1:**
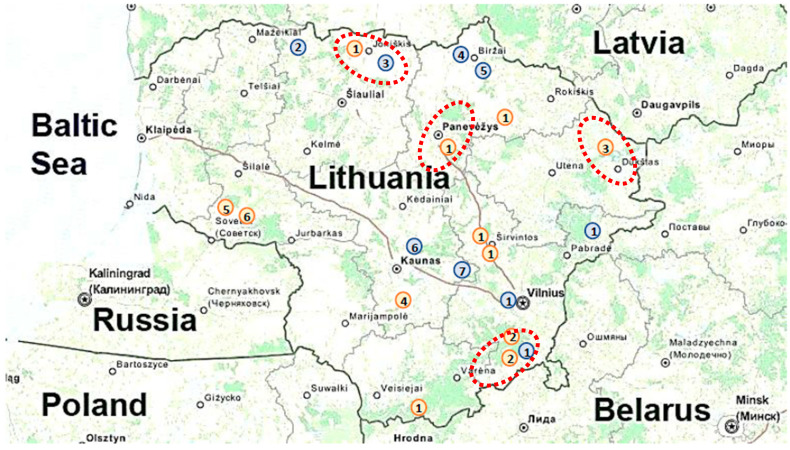
Sample collection sites in Lithuania, 2021. Orange circles represent DWV-B, and blue circles represent DWV-A. The number in the circle indicates the genetic variant of the identified virus. The red dotted circle indicates co-infestation locations.

A total of 12 good-quality DWV-B *RdRp* gene sequences obtained in this study were included in the genetic analysis. Sequence analysis revealed the presence of six DWV-B genotypes (with a difference at eight nucleotide positions and three parsimony-informative sites) in Lithuania. The distribution of genotypes across Lithuania is shown in Figure 1.

**Figure 2:**
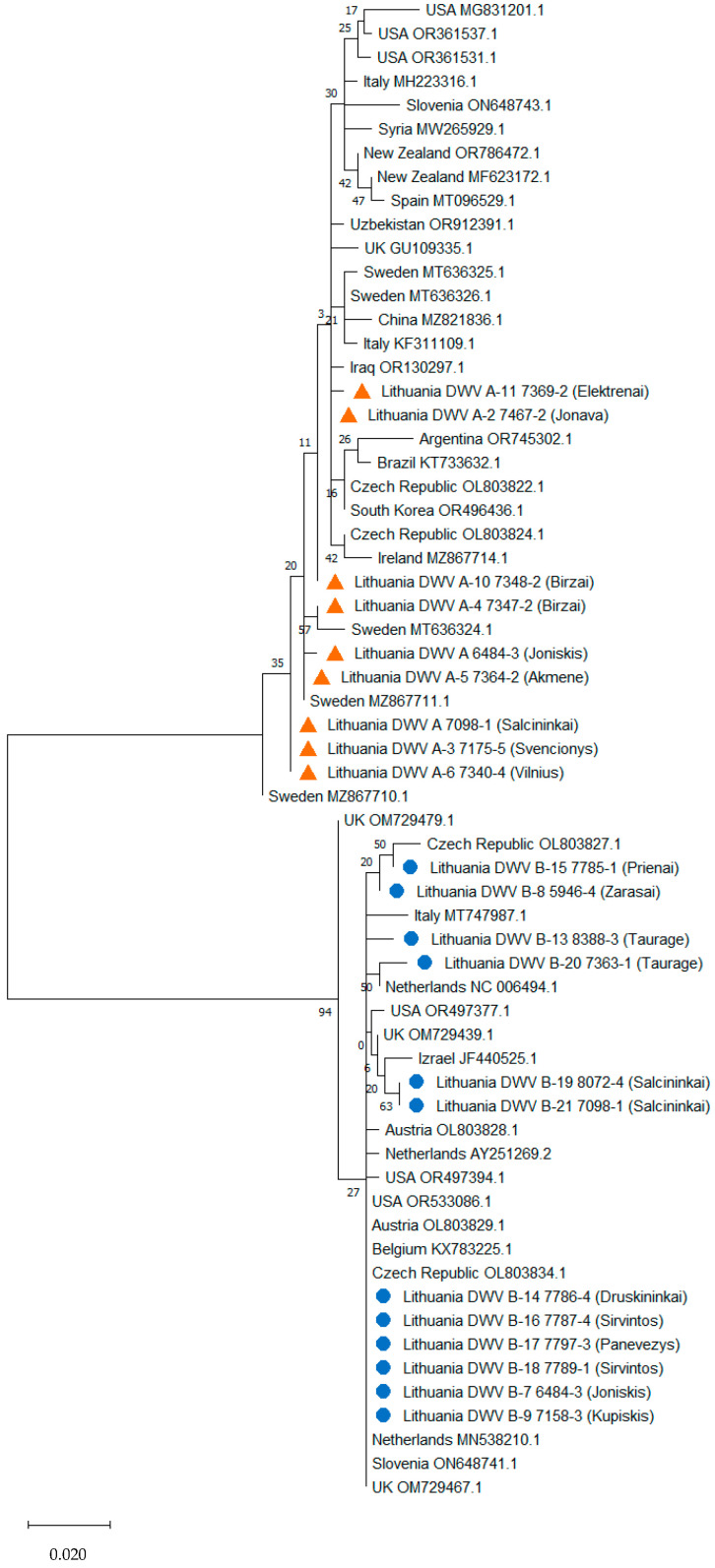
Maximum likelihood phylogenetic tree for the partial *RdRp* region of DWV-B and DWV-A. The phylogenetic tree was created using the Tamura–Nei model [40] and bootstrap analysis of 1000 replicates. Samples sequenced in the present study are marked (DWV-A orange triangle, DWV-B blue circle). The analysis involved 63 nucleotide sequences.

To analyse the genetic diversity of DWV-B strains, we included in the phylogenetic analysis seventeen GenBank sequences of DWV-B representing a group of sequences from the same region and the same identity percentage. The selected sequences were from the Netherlands (*n* = 3), Czech Republic (*n* = 2), Italy (*n* = 1), Israel (*n* = 1), Austria (*n* = 2), Belgium (*n* = 1), the United Kingdom (UK) (*n* =3), the United States of America (USA) (*n* = 3), and Slovenia (*n* = 1), and their identity to Lithuanian sequences ranged from 100% to 98.31% (Figure 2). The most common in Lithuania genotype 1 matched sequences from the Netherlands, Slovenia, UK, USA, Czech Republic, Austria, and Belgium, while other sequences from the Netherlands, Czech Republic, Italy, Austria, USA, UK, and Israel were unique and were assigned to different genotypes (Supplementary Table S5). Twenty variable sites were found when comparing the *RdRp* gene sequences of DWV-B isolates originating from Lithuania and other countries.

Nine good-quality DWV-A *RdRp* gene sequences obtained in this study were included in the phylogenetic analysis. All nine sequences were confirmed as DWV-A, and sequence analysis revealed the presence of seven DWV-A genotypes (with a difference in six nucleotide positions and three parsimony-informative sites) in Lithuania. The distribution of DWV-A genotypes across Lithuania is shown in Figure 1.

To analyse the genetic diversity and phylogenetic relations of DWV-A strains, twenty-five GenBank sequences of DWV-A representing a group of sequences from the same region and the same identity percentage were chosen. Sequences from Sweden (*n* = 5), Czech Republic (*n* = 2), Italy (*n* = 2), USA (*n* = 3), Spain (*n* = 1), Syria (*n* = 1), Ireland (*n* = 1), UK (*n* = 1), New Zealand (*n* = 2), Uzbekistan (*n* = 1), Iraq (*n* = 1), Argentina (*n* = 1), Brazil (*n* = 1), South Korea (*n* = 2), and China (*n* = 1) were included in the phylogenetic analysis, and their identity to Lithuanian sequences ranged from 100% to 97.64%. Thirty-one variable sites and thirty genotypes were found when comparing the Lithuanian sequences and those of other countries (Supplementary Table S6). Genotype 2 of the Lithuanian sequences matched sequences from Sweden, though all remaining sequences were assigned to different genotypes (Figure 2). The identity between DWV-A and DWV-B in the *RdRp* gene sequences was less than 87%. 

### 3.3. Phylogenetic Analysis of Coinfected Samples with DWV-A and DWV-B

For multilocus genetic analysis, five coinfected samples were selected and tested (Table 1, Figure 1). Not all gene fragments from all samples were successfully amplified (of the five DWV-A positive samples: Nlp = 5, Nvp3 = 5, Nhelicase = 4, Nrdrp = 3, and of the five DWV-B positive: Nlp = 4, Nvp3 = 5, Nhelicase = 4, Nrdrp = 4).

After amplification of the *helicase* gene, two DWV-A genotypes with three variable sites were detected. Three Lithuanian sequences were identical to two sequences from Turkey and highly similar (one nucleotide difference) to the sequence from Slovenia and one sequence from Turkey. Lithuanian sequences differed by six nucleotides from the DWV-A/DWV-B recombinant sequence from France (Supplementary Figure S3). 

Phylogenetic analysis of the DWV-B *helicase* gene revealed three genotypes with five variable sites. One Lithuanian sequence (77797-3) was found to be identical to the sequence from the Netherlands and a recombinant sequence from the USA. However, the other three Lithuanian sequences, two of which were identical, were unique and distinct from sequences from the other countries (Supplementary Figure S4).

Sequence analysis of the *LP* gene fragment of the DWV-A revealed four different genotypes and 37 variable sites. All analysed sequences were highly variable (59 variable sites), and four Lithuanian sequences had more than 89% similarity to DWV-A sequences obtained in other countries. One Lithuanian sequence (77797-3) had higher similarity to DWV sequences from Slovenia (5 variable sites) and the Netherlands (11 variable sites) and the recombinant sequence from the USA (6 variable sites); therefore, it was assigned to another cluster (Supplementary Figure S5). Sequence analysis of the *LP* fragment of the DWV-B also revealed four different genotypes and nine variable sites. Sequences were less variable (17 variable sites) compared to the *LP* gene of DWV-A and were assigned to the same cluster. However, the recombinant USA sequence was also highly similar to the analysed sequences (Supplementary Figure S6).

Sequence analysis of the *RdRp* fragment of the DWV-A revealed two different genotypes, and one sample was assigned to a different cluster (Supplementary Figure S7). Between two Lithuanian sequences, there were five variable sites, while between Lithuanian and other countries’ sequences assigned to DWV-A, there were ten variable sites. Both Lithuanian sequences were unique. The Kakugo virus was also included in phylogenetic analysis and showed 98% similarity to DWV-A sequences. The Lithuanian sample (77797-3) assigned to a separate cluster showed high similarity (four variable sites) to the DWV-B sequences from the UK and USA, and to the recombinant sequence from the USA. Sequence analysis of the *RdRp* fragment of the DWV-B revealed three different genotypes (six variable sites), and two sequences were identical (Supplementary Figure S8). As in previous sequences analysis, sample 77797-3 was the most genetically distinguished from other Lithuanian sequences and was identical to DWV-B sequences from the Netherlands and Austria and recombinant sequences from the Netherlands and the USA.

Lithuanian *VP3* gene sequences of DWV-A were divided into two clusters (Supplementary Figure S9). Two Lithuanian sequences were similar to DWV-A sequences from South Korea, China, New Zealand, the USA, and Sweden, while the other three sequences assigned to another cluster were identical to the recombinant from the USA and highly similar to the DWV-B sequences from the Netherlands and the recombinant from France. 

Sequence analysis of the *VP3* fragment of the DWV-B revealed three different genotypes (four variable sites). Three Lithuanian sequences were identical and highly similar to recombinants from the USA, Israel, and France. Sequences had six variable sites and 98–99% identity (Supplementary Figure S10). *VP3* gene phylogenetic analysis showed the highest similarity of Lithuanian sequences to DWV-A/B recombinant sequences obtained from the NCBI database. Sample number 77797-3 was genetically the closest to recombinant sequences according to the *LP*, *Vp3*, *Helicase*, and *RdRp* genes.

**Figure 3:**
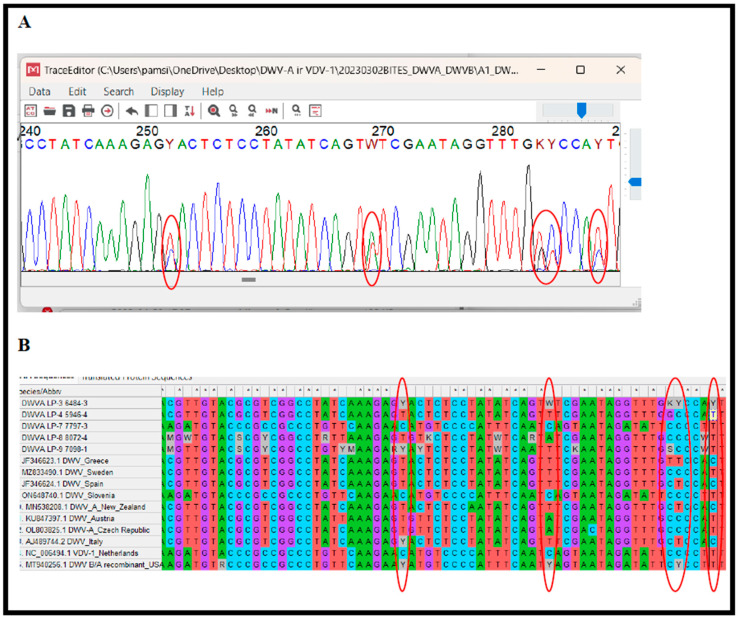
Infections of single and multiple DWV-A variants (*LP* gene) in *V. destructor* mites. Representative Sanger sequencing electrogram (forward) of PCR products revealed multiple DWV-A variants infections in *V. destructor* samples. Two peaks are present at five positions of the first sample (marked by red-6484-3) (**A**). Variable parts of sequences are marked by red (based on the first sample-6484-3) (**B**). Sequences were used in this study and obtained from NCBI database for alignment of multiple sequences and phylogenetic tree construction (**B**).

Examination of the Sanger sequencing electrograms demonstrated that *V. destructor* mites could also be infected by either a single or multiple DWV-A and DWV-B variants (Figure 3A). Multiple peaks were mainly present at electrograms of the *LP* gene in forward sequencing reactions. Double peaks were noticed in the most variable parts of the sequences obtained in this study and sequences from the NCBI database (Figure 3B). However, it is still possible that multiple peaks could be sequencing artifacts; therefore, a reverse sequencing reaction could be useful to confirm the results in further research.

## 4. Discussion

This study is the first report on the molecular characterization of mite-borne pathogens hosted by honeybees (*Apis mellifera*) in Lithuania. The aim of this study was to determine which DWV genotype is the most widespread in Lithuanian apiaries and in vectors carrying the virus and to evaluate the diversity of virus strains defined in infected *Varroa destructor* mites.

The deformed wing virus (DWV) from a largely unknown pathogen of honeybees has become one of the most well-known and widespread pathogens in the world. The rise in DWV’s prevalence and the growing interest in its genetic variability and pathogenicity are solely associated with the Varroa mite (*V. destructor*), the ectoparasite, which is well known as an efficient virus vector for virus transmission between honeybees [10,28]. Vertical and horizontal transmission routes of the virus lead to covert infections with no obvious symptoms or infections with clinical symptoms, which can lead to the rapid collapse of the entire bee colony [10,31,37,41]. Horizontal transmission by the *V. destructor* mite is likely to develop more virulent host–parasite relationships, whereas vertical transmission tends to favor more benign relationship diversity, which is important for the severity of DWV infections [10,31,42,43]. The newly emerged DWV-B is considered more virulent than the previously identified DWV-A. Having recently appeared, DWV-B can replicate in mites [11,31,44], which suggests that these strains would rapidly increase in abundance. An increasing number of Varroa mites, along with various biotic and abiotic factors and the conditions of the hosts, are likely to influence the DWV variant population and the levels of viral disease expression in different colonies and in different seasons [31,33,37,43,45].

In this study, 73 apiaries from 37 territories of Lithuania were investigated during the summer of 2021. Samples of covered broods were collected from 413 bee colonies. Infection of *V. destructor* mites was found in 63% of apiaries and 30% of bee colonies. The collected number of mites varied from 1 to 322 in hives infested with mites. A total of 1250 *V. destructor* mites were collected. Studies made in other countries also showed similar results. The prevalence of *V. destructor* mites in Turkey was 41% (84/204 colonies) [46]; in New Zealand, it was 27.8% (178/641 apiaries) [47]; in Belgium, about 72% of apiaries were infected with *V. destructor* mites [48]; and in China, 33% of brood cells were infected [49].

Most of the studies are concentrated on research on DWV-A and DWV-B genetic variants, which are found in adult bees or their broods, since DWV can be detected in all developmental stages and castes of bees. In this study, we investigated bee broods showing infection of DWV-B and DWV-A in 31.5% (23/73) and 24.7% (18/73) of apiaries, respectively. However, our main task was to investigate *V. destructor* mites, which can be vectors of both viruses. DWV-A was detected in 12.9% (16/124) of *V. destructor* mites, and DWV-B was detected in 56.5% (70/124) of samples collected in Lithuania. For sequence analysis, we used the *RdRp* gene, which is considered a good marker for studies concerning RNA virus classification and evolution. Sequence analysis revealed the presence of six DWV-B genotypes and seven genotypes of DWV-A in Lithuania. Due to the insufficient number of sequenced samples, we did not observe a geographical relationship between the prevalence of different virus variants in Lithuania. Phylogenetic analysis showed that most of the DWV-A sequences identified in Lithuania are close to the Swedish sequences, while the majority of DWV-B coincided with a large part of the sequences from European countries and the USA.

Coinfected samples were additionally investigated by using the *LP*, *Vp3*, *Helicase*, and *RdRp* genes. Multilocus analysis showed high nucleotide diversity of the investigated viruses and high similarity of strains with recombinant viruses isolated from other countries. Our analysis shows evidence and a high possibility of recombination in the *LP*-coding region. One investigated sample was highly similar to the recombinant virus from the USA (MT940254.1) according to all four genes and differed from other amplified sequences. There are several studies which have observed an increase in the virulence of recombinant viruses between DWV-A and DWV-B, and according to their reports, such recombinants would be adapted to being vectored by the mite [32,50] Mordecai et al. (2016) suggested that the emergence of recombinant variants may be the result of the DWV adaptation and evolutionary progress to a decrease in virulence and virus transmission optimization by Varroa mites in the honeybee population [51]. Recently, several recombinants issued from the A, B, and C types have been reported by McMahon et al. (2016), and based on research conducted in France, the minimal prevalence of recombinants could be around 85% [52].

Recombination is an important strategy for viruses which helps them to adapt to new environmental conditions and hosts and replicate more effectively. Viral recombination also serves to highlight the strongest strain-specific traits, so cooperation and self-regulation among viral strains can be suppressed. For this reason, recombinant viruses are also considered more virulent compared to their original genome counterparts [44]. Although recombination events around the world have been observed before [33,52], the recombination events of DWVs in Lithuania have never been described. Therefore, this study gave us new information not only about DWV-A and DWV-B prevalence and genetic diversity in Lithuania, but also about potentially detected recombinant viruses. Future studies with larger numbers of samples are required to confirm and discover more recombination cases, since the emergence of recombinants may contribute to the high levels of virus genetic diversity and viral adaptability to host in DWVs, which can have an impact on successful beekeeping.

The results of our study showed that the DVW-A and DWV-B virus strains and their presumably identified recombinants in Lithuanian apiaries can pose a threat to the health of bees, especially in the case of a high prevalence of varroa mites in hives. Although colonies can appear healthy, viral infections may be asymptomatic, and one or more adverse factors such as varroosis, weather conditions, food shortages, environmental chemicals, or other pathogens may cause these infections to become symptomatic and fatal. If viral infections are not very common and bees are not showing any obvious symptoms, the viral infection rate can be managed or reduced by controlling the spread of varroa mites in hives. Additionally, it is important to ensure that bees are able to reach a greater variety of honey plants and are kept in a healthy environment with a low pollution level and as few chemicals as possible [10,44]. Proper maintenance of honeybee hives, including good beekeeping practices, also needs to be improved. Good beekeeping management, new technologies, bee breeding, and transportation allow bees to be kept in a greater range of environments and decrease the possibility of infections [10].

## 5. Conclusions

This study has shown that DWV-A, DWV-B, and possibly detected DWV-A/B recombinants were prevalent in 2021 in different regions of Lithuania. High number of *V. destructor* mites were found in honeybee broods and were infected with both variants of DWV. The DWV-B infection rate in mites was higher compared with DVW-A. DWV-B (and DWV-A/B recombinants) may be better adapted for transmission by *V. destructor* than DWV-A.

Virus recombination might also give rise to novel variants of high virulence and encourage higher honeybee mortality.

Future research should consider the potential effects of new virus strains and investigate whether a new variant of the DWV has developed to establish the invasion risk level. Further studies should include more detailed analyses of bee broods and adult bees to determine the relationship between mite-borne virus strains and the level of bee infection with different virus strains. A genotype’s prevalence on the basis of a small region of the viral genome does not capture the total genetic diversity of a viral population and may obscure the role that recombination itself plays in competitive interactions among co-infecting viral genotypes. Therefore, we will consider conducting a more comprehensive analysis of DWV-A/B recombinants by using larger regions of the studied genes and a higher number of test samples. This approach would allow us to more accurately assess the potential extent of virus recombination, monitor changes over time, and evaluate the impact on bee health.

## Figures and Tables

**Table 1:** DWV-A and DWV-B samples used for phylogenetic analyses of four gene fragments.

	DWV-A				DWV-B			
	Amplification Fragment, Analysed Product Size (bp)							
Sample	*RdRp*	*Helicase*	*LP*	*VP3*	*RdRp*	*Helicase*	*LP*	*VP3*
6484-3	331	283	533	281	-	-	-	277
5946-4	-	283	533	281	345	208	363	277
7797-3	331	-	533	281	345	208	363	277
8072-4	331	283	533	281	345	208	363	277
7098-1	-	283	533	281	345	208	363	277

## Data Availability

The original contributions presented in the study are included in the article/supplementary material, further inquiries can be directed to the corresponding authors.

## Supplementary Materials

The following supporting information can be downloaded at: https://www.mdpi.com/article/10.3390/microorganisms12091884/s1. Reference [53] are cited in the supplementary materials.
