# Supplementary material for: Molecular Characterization and Phylogenetic Analysis of Honeybee (Apis mellifera) Mite-Borne Pathogen DWV-A and DWV-B Isolated from Lithuania
Source: Microorganisms. 2024 Sep 13;12(9):1884. doi: 10.3390/microorganisms12091884 (PMC11434569; doi:10.3390/microorganisms12091884)
Supplement: Supplementary file 1 [file microorganisms-12-01884-s001.zip › microorganisms-3108583-supplementary.pdf]

Supplementary Table S1. Varroa mites collected from apiaries located in different territories of Lithuania.

Nr.	Territories	Apiaries	Infected apiaries	Honey bee colonies	Infected colonies	*V. destructor* mites
**1**	Akmene	3	1	10	4	119
**2**	Alytus	2	1	12	2	35
**3**	Anyksciai	1	0	6	0	0
**4**	Birzai	2	2	7	6	63
**5**	Druskininkai	2	2	12	4	11
**6**	Elektrenai	1	1	6	2	3
**7**	Ignalina	2	2	12	3	5
**8**	Jonava	2	2	12	7	24
**9**	Joniskis	1	1	6	3	24
**10**	Jurbarkas	1	1	6	1	1
**11**	Kaisiadorys	2	1	12	1	5
**12**	Kaunas	1	0	6	0	0
**13**	Kelme	2	2	12	2	11
**14**	Kupiskis	2	2	12	6	9
**15**	Lazdijai	2	1	12	3	9
**16**	Marijampole	2	0	12	0	0
**17**	Moletai	3	1	18	1	2
**18**	Pagegiai	1	1	6	6	413
**19**	Pakruojis	2	1	12	1	1
**20**	Panevezys	3	1	18	5	100
**21**	Pasvalys	2	1	9	1	1
**22**	Prienai	2	1	12	6	26
**23**	Radviliskis	2	0	12	0	0
**24**	Raseiniai	2	2	12	3	10
**25**	Rokiskis	2	2	12	5	12
**26**	Sakiai	3	1	18	2	3
**27**	Salcininkai	2	2	12	7	242
**28**	Siauliai	2	1	12	2	2
**29**	Silale	2	2	11	4	8
**30**	Sirvintos	3	2	18	8	20
**31**	Svencionys	2	2	12	8	8
**32**	Taurage	1	1	4	4	39
**33**	Trakai	2	1	12	1	1
**34**	Utena	2	1	12	3	14
**35**	Varena	2	0	12	0	0
**36**	Vilnius	3	2	12	6	16
**37**	Zarasai	2	2	12	7	13
	**Total**	**73**	**46 (63%)**	**413**	**124 (30%)**	**1250**

Supplementary Table S2. The oligo primer pairs and probes selected for RT-qPCR, which were specific to *CRPV* capsid region of the DWV-A and DWV-B to detect the infection of particular pathogen.

Target	Genome region	Primer, probe name	Sequence (5' –3')	Primer, probe position[a]	Product length (bp)	Reference
DWV-A	*CRPV*-capsid	DWV-A for	GCGGCTAAGATTGTAAATTG	4245-4264	72	[53]
		DWV-A rev	GTGACTAGCATAACCATGATTA	4295-4316		
		DWV-A probe	FAM-CCTTGACCAGTAGACACAGCATC-TAMRA	4266-4288		
DWV-B	*CRPV*-capsid	DWV-B for	GGTCTGAAGCGAAAATAG	4218-4235	73	
		DWV-B rev	CTAGCATATCCATGATTATAAAC	4268-4290		
		DWV-B probe	FAM-CCTTGTCCAGTAGATACAGCATCACA-TAMRA	4241-4266		

[a]Primers and probes positions refer to those in the published complete DWV-A and DWV-B sequences (GenBank accession number AY292384.1 [16] and AY251269.2 [14].

Supplementary Table S3. The oligo primer pairs selected for sequencing, which were specific to *RdRp* region of the selected DWV-A and DWV-B mites samples.

Target	Genome region	Primer, probe name	Sequence (5' –3')	Primer position[b]	Product length (bp)	Reference
DWV-A	RNA-dependent RNA polymerase (*RdRp*)	DWV-F1a	GGAAACATCTGGAATTAGCGACAA	8476-8499	339	[33]
		DWV-VDV 7aR	AATCCGTGAATATAGTGTGAGG	8793-8814		
DWV-B	RNA-dependent RNA polymerase (*RdRp*)	VDV-F1a	GAAAACATTTGGAATTAGCAACGAC	8454-8478	339	
		DWV-VDV 7aR	AATCCGTGAATATAGTGTGAGG	8771-8791		

[b]Primers positions refer to those in the published complete DWV-A and DWV-B sequences (GenBank accession number AY292384.1 [16] and AY251269.2 [14]).

Supplementary Table S4. Primer sequences, positions, thermal protocols and amplicon product size for four genomic fragments of DWV-A and DWV-B.

**Target**	**Primer name and position**	**Sequence (5' –3')**	**Amplification program 40 cycles**	**Product length (bp)**
**DWV-A**	Lp _F1153<br>Lp_ B1806	ATTAAAAATGGCCTTTAGTTG<br>CTTTTCTAATTCAACTTCACC	20 min 50°C<br>2 min 95°C<br>30 s 94°C<br>45 s 55°C<br>45 s 72°C<br>10 min 72°C	653
	VP3_DWVF1<br>VP3_DWVB1	CCTGCTAATCAACAAGGACCTGG<br>CAGAACCAATGTCTAACGCTAACCC	20 min 50°C<br>2 min 95°C<br>30 s 94°C<br>30 s 55°C<br>30 s 72°C<br>10 min 72°C	355
	Helicase_6285F<br>Helicase_6693R	GAGCGTACACTATGGTCAGA<br>GTTCACGACGCTTACTACAC		409
	RdRp_F15(9247)<br>RdRp_B23(9697)	TCCATCAGGTTCTCCAATAACGGA<br>CCACCCAAATGCTAACTCTAAGCG		450
**DWV-B**	Lp_1_1520F<br>Lp_1_1998R	AAGAAAGTGAAACGGGTGGC<br>ATTAAGCGCGCCAATTCCTT	20 min 50°C<br>2 min 95°C<br>30 s 94°C<br>30 s 60°C<br>30 s 72°C<br>10 min 72°C	437
	VP3_1_3707<br>VP3_1_4089	CAAGGACCCGGCAAAGTAAG<br>CCATCACGGCAGCGATTAAA		383
	Heli_1_6428<br>Heli_1_6745	TATGCAGCAGGAATGAACGC<br>TGTAGAACGCTCGTGGACAT		318
	RdRp_2_9343<br>RdRp_2_9777	CGTGCTAGTTTGTTACGGTGA<br>ACATCCATTTCTTCCCATGTGA		435

* DWV primers taken from Manley R. A. (2017), amplification conditions determined in this study by experimental methods [13].

Supplementary Table S5. DWV-B genotypes of studied *Varroa destructor* mites' samples based on the partial *RdRp* region analysis.

Sample	Genetic variant	Nucleotide position of variable sites																			
		16	18	36	42	60	69	78	87	93	99	108	123	156	183	201	210	216	245	252	282
**Lithuania DWV B-13 8388-3 (Taurage)**	6	G	C	C	C	A	G	A	G	C	T	G	T	C	T	A	T	G	A	A	A
**Lithuania DWV B-14 7786-4 (Druskininkai)**	1	T	.	.	.	.	.	.	.	.	.	C	.	.	.	.	.	.	.	.	.
**Lithuania DWV B-15 7785-1 (Prienai)**	4	T	.	.	T	.	.	.	.	T	.	C	.	.	.	.	.	.	.	.	.
**Lithuania DWV B-17 7797-3 (Panevezys)**	1	T	.	.	.	.	.	.	.	.	.	C	.	.	.	.	.	.	.	.	.
**Lithuania DWV B-16 7787-4 (Sirvintos)**	1	T	.	.	.	.	.	.	.	.	.	C	.	.	.	.	.	.	.	.	.
**Lithuania DWV B-18 7789-1 (Sirvintos)**	1	T	.	.	.	.	.	.	.	.	.	C	.	.	.	.	.	.	.	.	.
**Lithuania DWV B-19 8072-4 (Salcininkai)**	2	T	.	T	.	.	A	.	.	.	.	C	.	.	.	.	.	.	.	.	.
**Lithuania DWV B-20 7363-1 (Taurage)**	5	T	T	T	.	.	.	.	.	.	.	C	.	.	.	.	.	.	.	G	.
**Lithuania DWV B-7 6484-3 (Joniskis)**	1	T	.	.	.	.	.	.	.	.	.	C	.	.	.	.	.	.	.	.	.
**Lithuania DWV B-21 7098-1 (Salcininkai)**	2	T	.	T	.	.	A	.	.	.	.	C	.	.	.	.	.	.	.	.	.
**Lithuania DWV B-8 5946-4 (Zarasai)**	3	T	.	.	.	.	.	.	.	T	.	C	.	.	.	.	.	.	.	.	.
**Lithuania DWV B-9 7158-3 (Kupiskis)**	1	T	.	.	.	.	.	.	.	.	.	C	.	.	.	.	.	.	.	.	.
**Austria OL803828.1**	6	T	.	.	.	.	.	.	.	.	C	C	.	.	.	.	.	.	.	.	.
**Austria OL803829.1**	1	T	.	.	.	.	.	.	.	.	.	C	.	.	.	.	.	.	.	.	.
**Belgium KX783225.1**	1	T	.	.	.	.	.	.	.	.	.	C	.	.	.	.	.	.	.	.	.
**Czech Republic OL803827.1**	7	T	.	.	T	.	.	.	.	.	.	C	.	.	.	.	.	A	.	.	.
**Czech Republic OL803834.1**	1	T	.	.	.	.	.	.	.	.	.	C	.	.	.	.	.	.	.	.	.
**Italy MT747987.1**	8	T	.	.	.	.	.	G	.	.	.	C	.	T	.	G	.	.	.	.	.
**Izrael JF440525.1**	9	T	.	T	.	.	.	.	.	.	.	C	A	.	.	.	.	.	T	.	.
**Netherlands AY251269.2**	10	T	.	.	.	.	.	.	.	.	.	C	.	.	.	.	.	.	.	.	G
**Netherlands MN538210.1**	1	T	.	.	.	.	.	.	.	.	.	C	.	.	.	.	.	.	.	.	.
**Slovenia ON648741.1**	1	T	.	.	.	.	.	.	.	.	.	C	.	.	.	.	.	.	.	.	.
**Netherlands NC 006494.1**	11	T	.	.	.	.	.	.	.	.	.	C	.	.	.	.	.	.	.	G	.
**UK OM729439.1**	12	T	.	?	.	.	.	.	.	.	.	C	.	.	.	.	.	.	.	.	.

**UK OM729467.1**	1	T	.	.	.	.	.	.	.	.	.	C	.	.	.	.	.	.	.	.	.
**UK OM729479.1**	13	T	.	.	.	.	.	.	A	.	.	C	.	.	C	.	.	.	.	.	.
**USA OR497377.1**	14	T	.	.	.	G	.	.	.	.	.	C	.	.	.	.	.	.	.	.	.
**USA OR497394.1**	15	T	.	.	.	.	.	.	.	.	.	C	.	.	.	.	C	.	.	.	.
**USA OR533086.1**	1	T	.	.	.	.	.	.	.	.	.	C	.	.	.	.	.	.	.	.	.

Supplementary Table S6. DWV-A genotypes of studied *Varroa destructor* mites' samples based on the partial *RdRp* region analysis.

**Sample**	**Genetic variant**	**Nucleotide position of variable sites**																														
		13	18	36	55	57	69	75	77	78	81	84	85	87	102	108	135	165	168	181	183	186	189	198	204	213	214	249	255	258	264	270
**Lithuania DWV A 6484-3 (Joniskis)**	3	G	A	T	T	G	G	T	T	G	T	T	T	A	G	T	A	T	G	G	C	T	G	T	A	C	T	A	G	A	A	G
**Lithuania DWV A 7098-1 (Salcininkai)**	1	.	.	.	.	.	A	.	.	.	.	.	.	.	.	.	.	.	.	.	.	.	.	.	.	T	.	.	.	.	.	.
**Lithuania DWV A-10 7348-2 (Birzai)**	5	.	.	.	.	.	A	.	.	.	.	.	.	.	.	.	.	.	.	.	.	.	.	C	.	.	.	.	.	.	.	.
**Lithuania DWV A-11 7369-2 (Elektrenai)**	7	.	.	.	.	.	A	.	.	.	.	.	.	.	.	.	G	.	.	T	.	.	.	C	.	.	.	.	.	.	.	.
**Lithuania DWV A-2 7467-2 (Jonava)**	6	.	.	.	.	.	A	.	.	.	.	.	.	.	.	.	G	.	.	.	.	.	.	C	.	.	.	.	.	.	.	.
**Lithuania DWV A-3 7175-5 (Svencionys)**	1	.	.	.	.	.	A	.	.	.	.	.	.	.	.	.	.	.	.	.	.	.	.	.	.	T	.	.	.	.	.	.
**Lithuania DWV A-4 7347-2 (Birzai)**	4	.	.	.	.	.	A	.	.	.	.	.	.	.	.	.	.	C	.	.	.	.	.	.	.	.	.	.	.	.	.	.
**Lithuania DWV A-5 7364-2 (Akmene)**	2	.	.	.	.	.	A	.	.	.	.	.	.	.	.	.	.	.	.	.	.	.	.	.	.	.	.	.	.	.	.	.
**Lithuania DWV A-6 7340-4 (Vilnius)**	1	.	.	.	.	.	A	.	.	.	.	.	.	.	.	.	.	.	.	.	.	.	.	.	.	T	.	.	.	.	.	.
**Argentina OR745302.1**	9	.	G	.	.	.	A	.	.	A	.	.	G	G	.	.	G	.	.	.	.	.	.	.	.	T	.	.	.	.	.	.
**Brazil KT733632.1**	10	.	G	.	.	.	A	.	.	.	.	.	.	.	A	.	G	.	.	.	.	.	.	C	.	T	.	.	.	.	.	.
**China MZ821836.1**	11	.	.	.	C	.	A	.	.	.	.	.	.	.	.	.	G	.	A	.	.	.	.	.	.	.	.	.	.	.	.	.
**Czech Republic OL803822.1**	8	.	.	.	.	.	A	.	.	.	.	.	.	.	.	.	G	.	.	.	.	.	.	C	.	T	.	.	.	.	.	.
**Czech Republic OL803824.1**	12	.	.	.	.	.	A	.	.	.	.	.	.	.	.	.	G	.	.	.	T	.	.	C	.	.	.	.	.	.	.	.
**Iraq OR130297.1**	13	.	.	.	.	.	A	.	.	.	.	.	.	.	.	.	G	.	.	.	.	.	.	C	G	.	.	.	.	.	.	.
**Ireland MZ867714.1**	14	.	.	.	.	.	A	.	.	.	A	.	.	.	.	.	.	.	.	.	T	.	.	C	.	.	.	.	.	.	.	.
**Italy KF311109.1**	15	.	.	.	.	.	A	.	.	.	.	.	.	.	.	.	G	.	.	.	.	.	A	.	.	.	.	.	.	.	.	.
**Italy MH223316.1**	16	.	.	.	.	.	A	.	.	.	.	.	.	.	.	.	G	.	.	.	.	.	.	C	.	.	C	.	.	.	.	A
**New Zealand MF623172.1**	17	.	.	C	.	.	A	.	.	.	.	.	.	.	.	.	G	.	.	.	.	C	.	C	.	.	C	.	.	.	.	.
**New Zealand OR786472.1**	18	.	.	C	.	.	A	.	.	.	.	.	.	.	.	.	G	.	.	.	.	.	.	C	.	.	C	.	.	.	.	.

**Syria MW265929.1**	19	.	.	.	.	.	A	C	C	.	.	.	.	.	.	.	G	.	.	.	.	.	.	C	.	.	C	.	.	.	.	.
**Slovenia ON648743.1**	20	A	.	.	.	A	A	A	.	.	.	.	.	.	.	.	.	.	.	.	.	.	.	C	.	.	C	.	.	.	.	.
**South Korea OR496436.1**	8	.	.	.	.	.	A	.	.	.	.	.	.	.	.	.	G	.	.	.	.	.	.	C	.	T	.	.	.	.	.	.
**Spain MT096529.1**	21	.	.	.	.	.	A	.	.	.	.	.	.	.	.	.	G	.	.	.	.	C	.	C	.	.	C	.	.	.	.	.
**Sweden MT636324.1**	22	.	.	.	.	.	A	.	.	.	.	.	.	.	.	.	.	C	.	.	.	.	.	.	.	.	.	G	.	.	G	.
**Sweden MT636325.1**	23	T	.	.	.	.	A	.	.	.	.	.	.	.	.	.	G	.	.	.	.	.	.	.	.	.	.	.	.	.	.	.
**Sweden MT636326.1**	24	.	.	.	.	.	A	.	.	.	.	.	.	.	.	.	G	.	.	.	.	.	.	.	.	.	.	.	.	.	.	.
**Sweden MZ867710.1**	25	.	.	.	.	.	A	.	.	.	.	.	.	.	.	.	.	.	.	.	.	.	.	.	.	T	.	.	A	G	.	.
**Sweden MZ867711.1**	2	.	.	.	.	.	A	.	.	.	.	.	.	.	.	.	.	.	.	.	.	.	.	.	.	.	.	.	.	.	.	.
**UK GU109335.1**	26	A	.	.	.	.	A	.	.	.	.	.	G	.	.	.	G	.	.	.	.	.	.	C	.	.	.	.	.	.	.	.
**USA MG831201.1**	27	T	.	.	.	.	A	.	.	.	.	A	.	.	.	.	G	C	.	.	.	.	.	.	.	.	C	.	.	.	.	A
**USA OR361531.1**	28	.	.	.	.	.	A	.	.	.	.	A	.	.	.	C	G	.	.	.	.	.	.	C	.	.	C	.	.	.	.	.
**USA OR361537.1**	29	A	.	.	.	.	A	.	.	.	.	A	.	.	.	.	G	.	.	.	.	.	.	C	.	.	C	.	.	.	.	.
**Uzbekistan OR912391.1**	30	.	.	.	.	.	A	.	.	.	.	C	.	.	.	.	G	.	.	.	.	.	.	C	.	.	.	.	.	.	.	.

Supplementary Figure S1. QIAxcel capillary electrophoresis image visualizes samples of *Varroa destructor* mites which been used for DWV-A infection confirmations by using *RdRp* region primers for fragments amplification. Framed samples have been selected for sequencing and phylogenetic analysis.

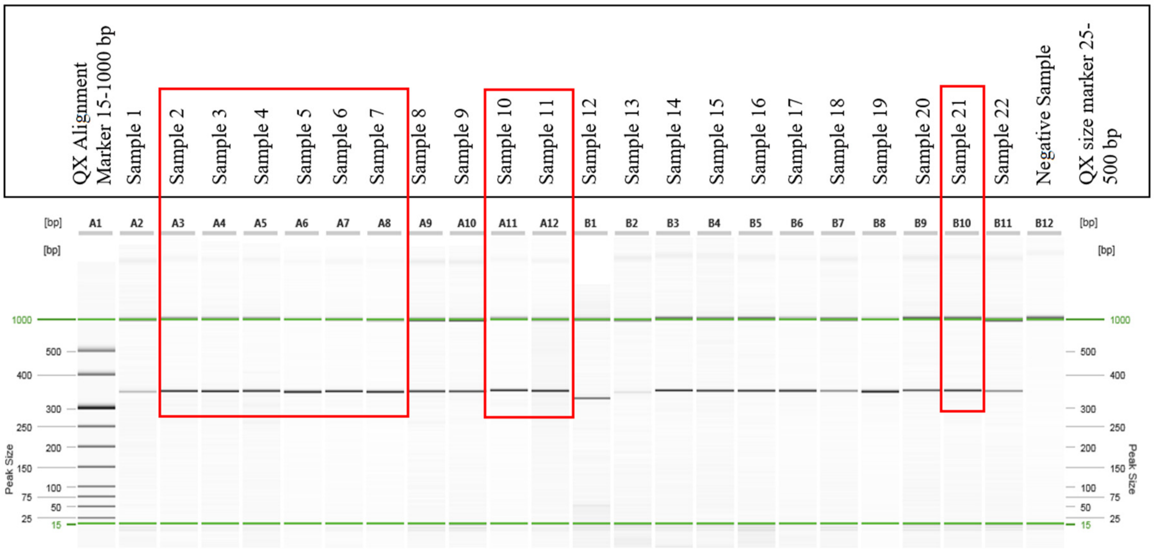

Supplementary Figure S2. QIAxcel capillary electrophoresis image visualize samples of *Varroa destructor* mites which been used for DWV-B infection confirmations by using *RdRp* region primers for fragments amplification. Framed samples have been selected for sequencing and phylogenetic analysis.

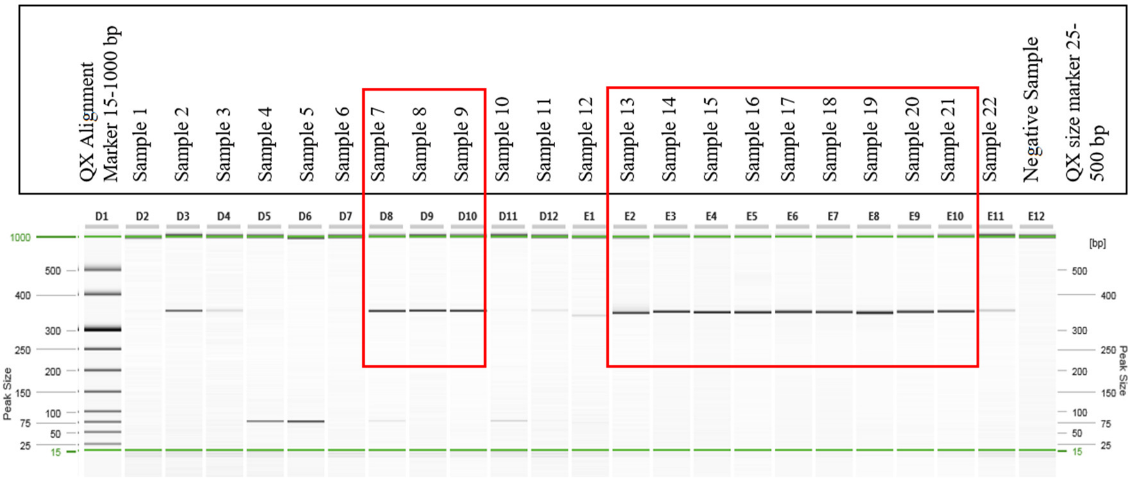

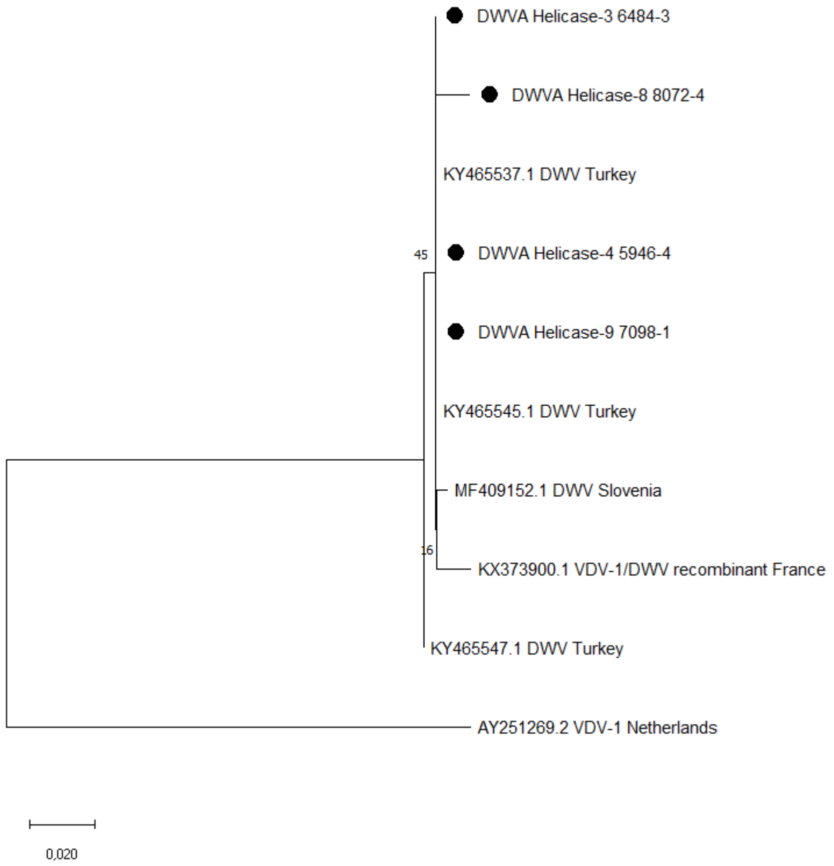

Supplementary Figure S3. Maximum Likelihood phylogenetic tree for the *helicase* gene region of DWV-A. The phylogenetic tree was created using the Tamura-Nei model [40] and bootstrap analysis of 1000 replicates. Samples sequenced in the present study are marked. For phylogenetic analyses were used 283 bp length sequences.

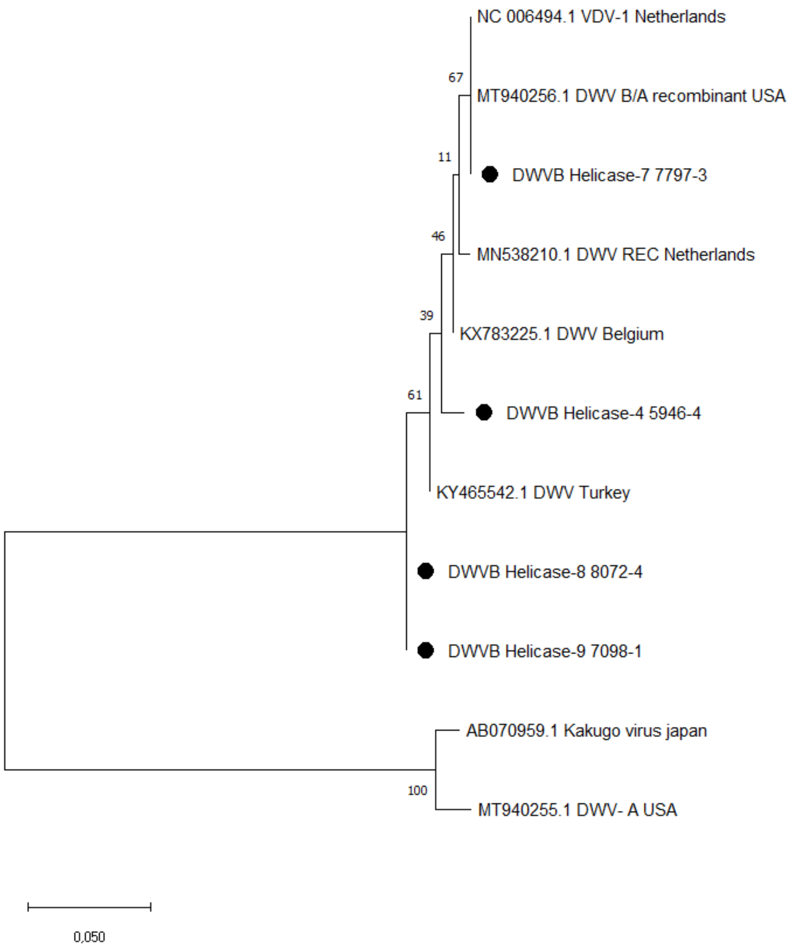

Supplementary Figure S4. Maximum Likelihood phylogenetic tree for the *helicase* gene region of DWV-B. The phylogenetic tree was created using the Tamura-Nei model [40] and bootstrap analysis of 1000 replicates. Samples sequenced in the present study are marked. For phylogenetic analyses were used 208 bp length sequences.

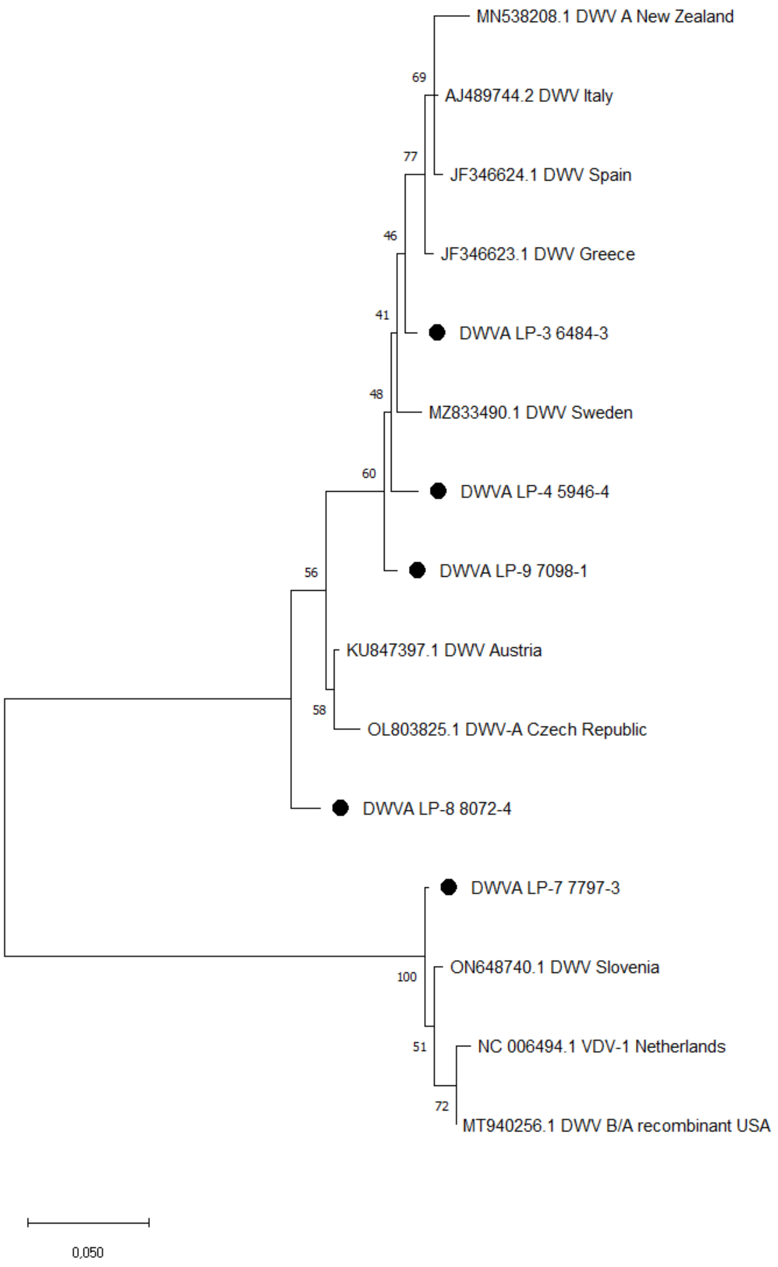

Supplementary Figure S5. Maximum Likelihood phylogenetic tree for the *LP* gene region of DWV-A. The phylogenetic tree was created using the Tamura-Nei model [40] and bootstrap analysis of 1000 replicates. Samples sequenced in the present study are marked. For phylogenetic analyses were used 533 bp length sequences.

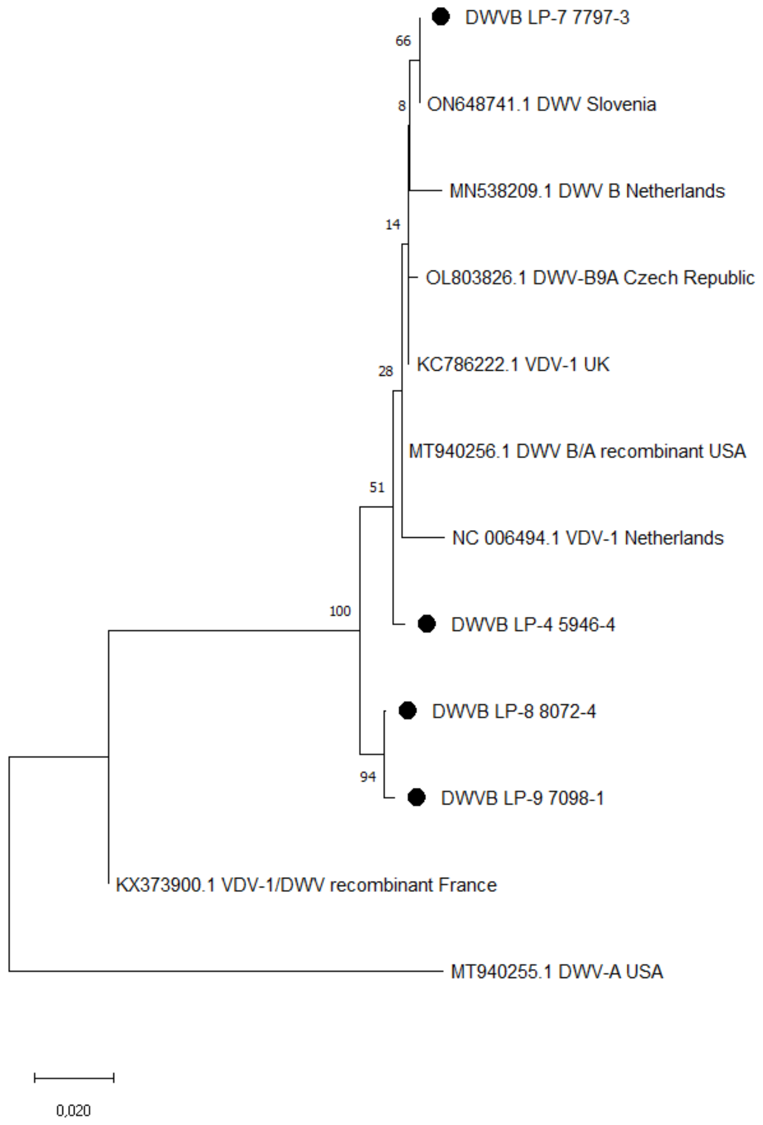

Supplementary Figure S6. Maximum Likelihood phylogenetic tree for the *LP* gene region of DWV-B. The phylogenetic tree was created using the Tamura-Nei model [40] and bootstrap analysis of 1000 replicates. Samples sequenced in the present study are marked. For phylogenetic analyses were used 363 bp length sequences.

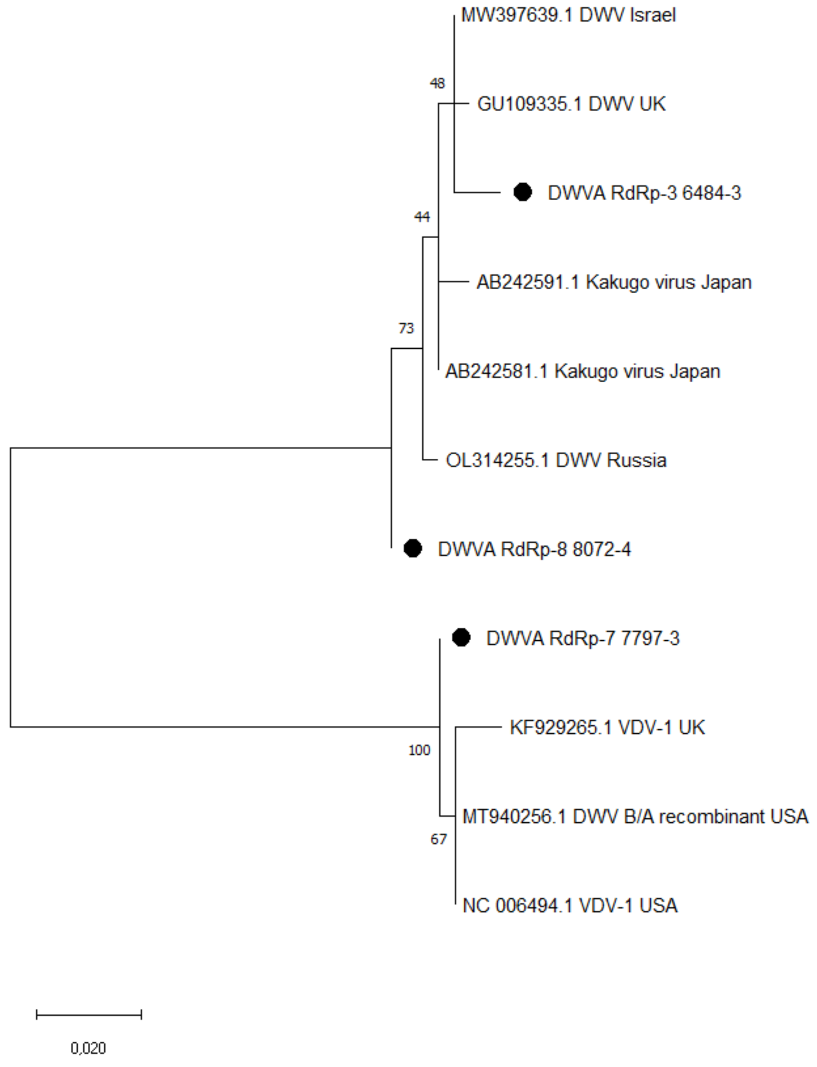

Supplementary Figure S7. Maximum Likelihood phylogenetic tree for the *RdRp* gene region of DWV-A. The phylogenetic tree was created using the Tamura-Nei model [40] and bootstrap analysis of 1000 replicates. Samples sequenced in the present study are marked. For phylogenetic analyses were used 331 bp length sequences.

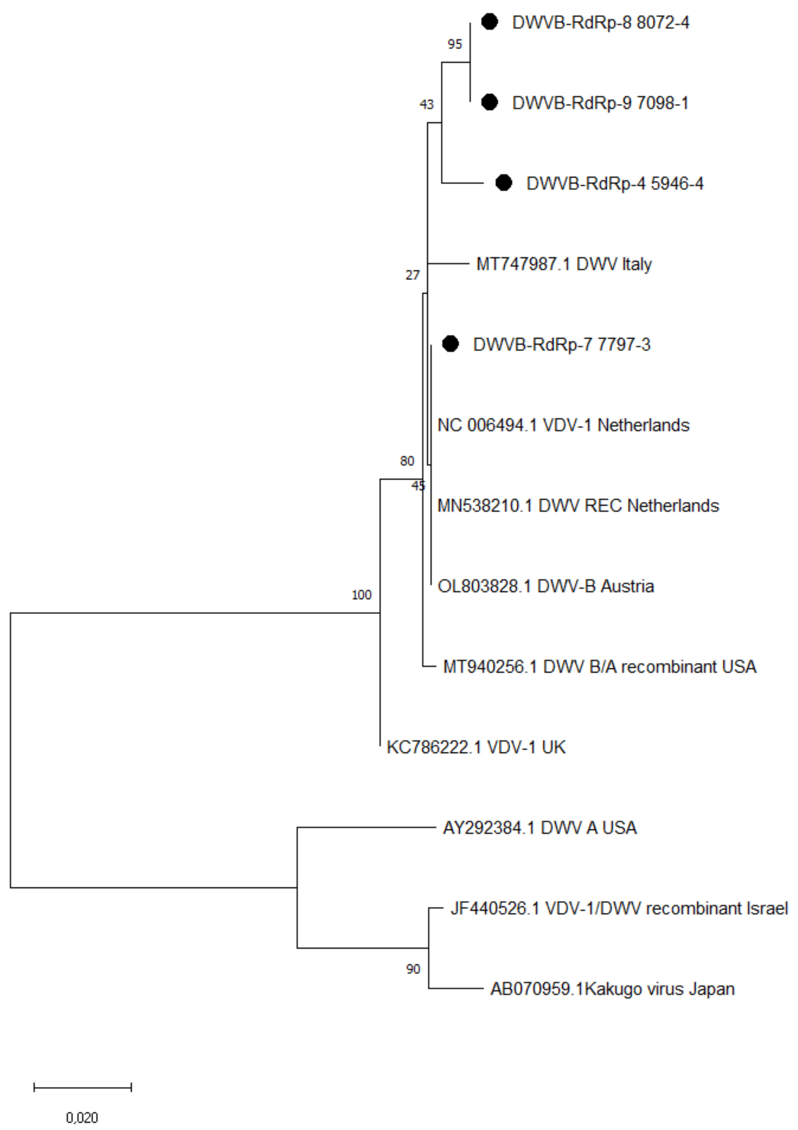

Supplementary Figure S8. Maximum Likelihood phylogenetic tree for the *RdRp* gene region of DWV-A. The phylogenetic tree was created using the Tamura-Nei model [40] and bootstrap analysis of 1000 replicates. Samples sequenced in the present study are marked. For phylogenetic analyses were used 345 bp length sequences.

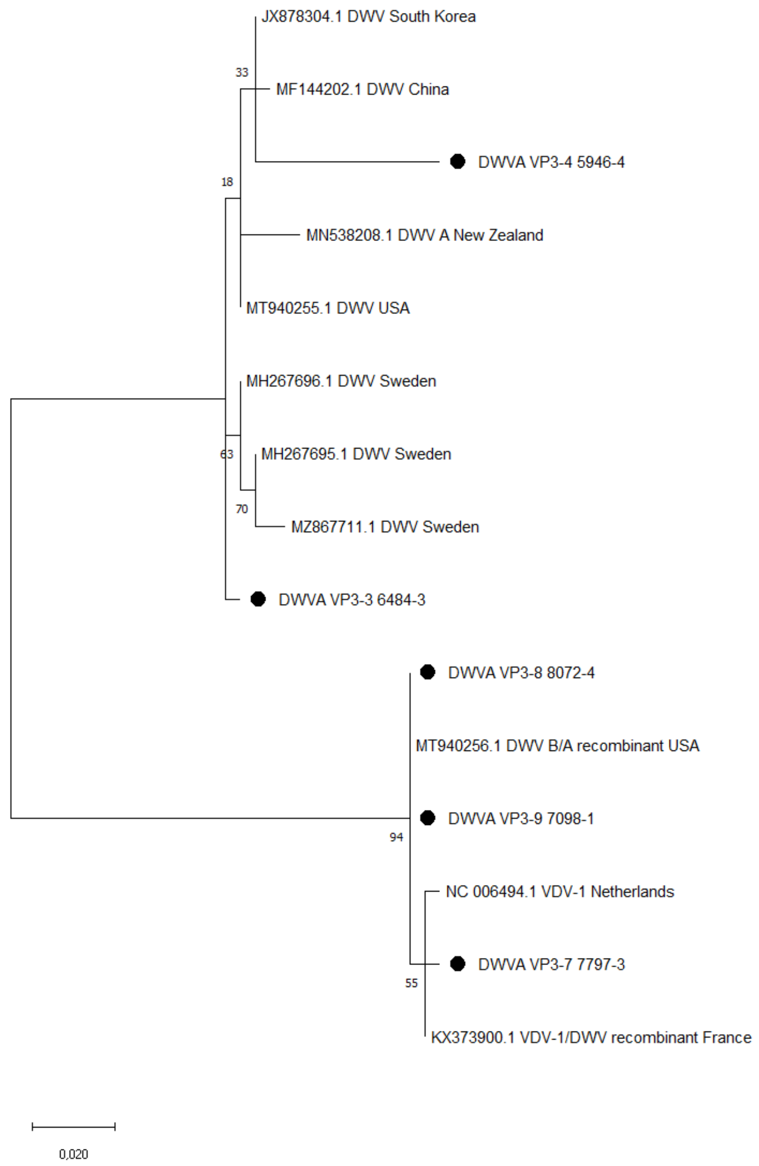

Supplementary Figure S9. Maximum Likelihood phylogenetic tree for the *VP3* gene region of DWV-A. The phylogenetic tree was created using the Tamura-Nei model [40] and bootstrap analysis of 1000 replicates. Samples sequenced in the present study are marked. For phylogenetic analyses were used 281 bp length sequences.

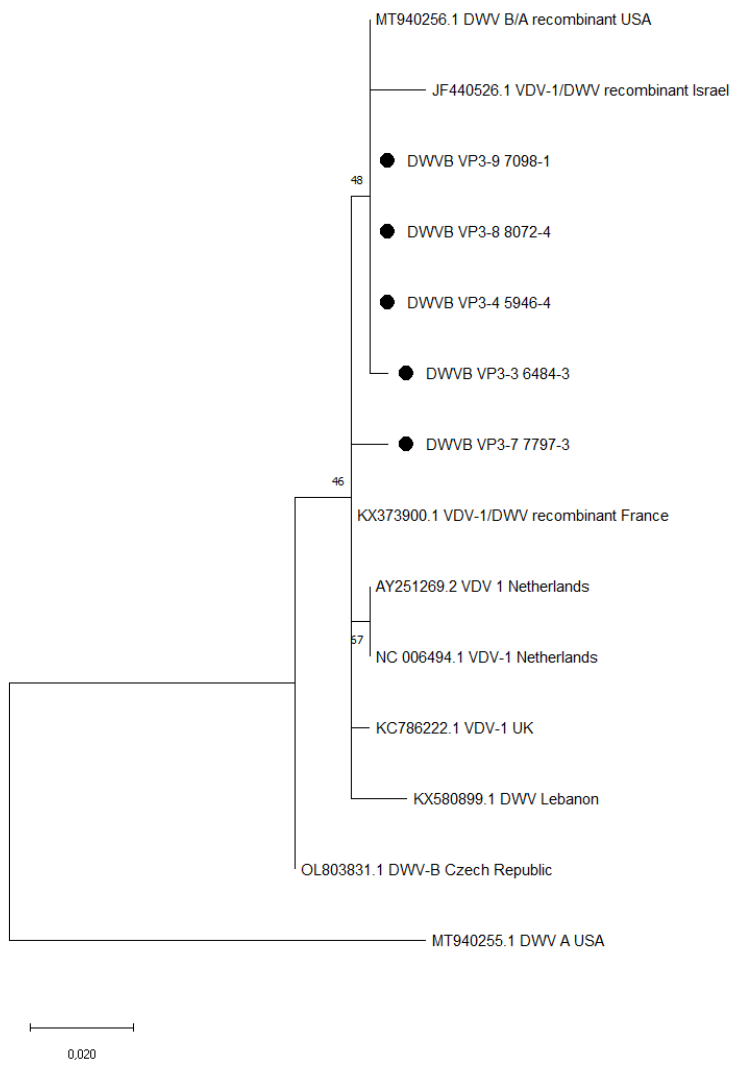

Supplementary Figure S10. Maximum Likelihood phylogenetic tree for the *VP3* gene region of DWV-B. The phylogenetic tree was created using the Tamura-Nei model [40] and bootstrap analysis of 1000 replicates. Samples sequenced in the present study are marked. For phylogenetic analyses were used 277 bp length sequences.
